# Wearable Tailored Passive Radiative Cooling Textile for Flexible Electronic Integration

**DOI:** 10.1002/advs.202524380

**Published:** 2026-03-18

**Authors:** Lung Chow, Jianpeng Zhang, Zehua Peng, Denglin Ye, Binbin Zhang, Jian Li, Wooyoung Park, Chentao Du, Xingcan Huang, Chun Ki Yiu, Yuze Qiu, Jingkun Zhou, Zhenlin Chen, Yuyu Gao, Weibin Zhu, Pengcheng Wu, Guangyao Zhao, Qiang Zhang, Yuan Guo, Zuowei Sun, Zhan Shen, Shuaiqi Ren, Sang Min Won, Jiyu Li, Xinge Yu

**Affiliations:** ^1^ Department of Biomedical Engineering City University of Hong Kong Hong Kong China; ^2^ Hong Kong Centre for Cerebro‐Cardiovascular Health Engineering Hong Kong China; ^3^ School of Biomedical Engineering Sun Yat‐sen University Shenzhen China; ^4^ Key Laboratory of Photonic‐Electronic Integration and Communication‐Sensing Convergence School of Information Science and Technology Southwest Jiaotong University Chengdu China; ^5^ Institute of Digital Medicine City University of Hong Kong Kowloon Hong Kong China; ^6^ Department of Electrical and Computer Engineering Sungkyunkwan University (SKKU) Suwon Republic of Korea

**Keywords:** radiative cooling, textile electronics, thermal‐optical regulation, wearable electronics

## Abstract

Textiles are ideal platforms for wearable electronics due to their inherent softness and superior thermophysiological comfort. However, conventional textiles prioritize wearer comfort at the cost of the stringent thermal‐optical demands imposed by embedded electronics, often sacrificing scalability, breathability, electronic integrability, device performance, and user safety. Here, we report a wearable tailored passive radiative cooling textile (WRCT) for seamless integration of flexible electronics. The WRCT was fabricated via a scalable, one‐step, and additive‐free wet‐spinning technique. Hierarchical phase inversion kinetics create microfibers with multiscale porosity and surface nodules, achieving solar reflectance (>95%) and mid‐infrared emissivity (0.96). This single‐material platform satisfies the conflicting requirements of wearing comfort and electronic functionality by providing breathability, flexibility, and passive daytime thermal management. These properties thermally decouple electronics from the skin and keep skin temperature below 41°C even under intense sunlight (500 W/m^2^) combined with a localized heat load equivalent to a heat flux of approximately 17 kW/m^2^ over a 142 mm^2^ area, simulating high‐power microcontrollers, conditions that cause low‐temperature burns within minutes on conventional textiles. By converting a commodity polymer into an advanced thermal‐optical regulator through a mature and scalable fiber‐production process, this textile establishes a practical, safe, and manufacturable foundation for reliable, all‐day wearable electronic systems.

## Graphical abstract

A scalable, additive‐free wearable tailored passive radiative cooling textile (WRCT) with hierarchically structured fibers delivers exceptional thermal management, combining >95% solar reflectance, 0.96 mid‐infrared emissivity, and ultralow thermal conductivity (0.041 W/m·K). This synergy maintains safe skin temperatures under extreme heat from electronics and solar radiation, ensuring reliable, comfortable operation of all‐day wearable electronics.

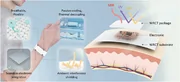

## 1 Introduction

Wearable electronics have emerged as a technology for continuous health monitoring [1, 2, 3, 4, 5, 6], human‐machine interfaces [7, 8, 9, 10, 11, 12, 13], motion tracking [14, 15], and personalized thermal regulation [16, 17, 18]. For truly all‐day wear, thermophysiological neutrality with minimal disruption of the skin's natural heat and moisture exchange is essential. Integrating soft electronics directly into textiles has thus become a central pursuit, fueling the rapid growth of electronic textiles (e‐textiles) [1, 10, 19, 20, 21]. Textiles represent humanity's most skin‐compatible interface, refined over millennia to be breathable, soft, lightweight, washable, and highly conformable [19]. Their hierarchical porosity ensures efficient air and water‐vapor transport, making them the ideal substrate for wearable systems that users will adopt long‐term.

Yet the practical deployment of textile‐based wearables remains fundamentally hindered by an unresolved conflict in thermal‐optical requirements. Human skin demands high breathability to enable rapid sweat evaporation and maintain comfort between 33°C and 37°C [22, 23]. In contrast, embedded electronics, driven by ongoing advancements that deliver increasingly powerful functionality, typically operate at much higher temperatures [17, 24] (>60°C), producing substantial Joule heat that must be dissipated while remaining thermally decoupled from the skin to prevent low‐temperature burns above 43°C during prolonged contact [25]. Simultaneously, reliable outdoor performance of photodetectors, sensors, antennas, and microcontrollers, etc. requires near‐perfect sunlight rejection to suppress photothermal noise, device overheating, and signal drift. To date, no conventional textile has simultaneously delivered high solar reflectance, strong radiative cooling, excellent breathability, and robust thermal decoupling of electronics from the skin while preserving seamless electronic integration, mechanical robustness, flexibility, washability, cost‐effectiveness, and industrial scalability.

**FIGURE 1:**
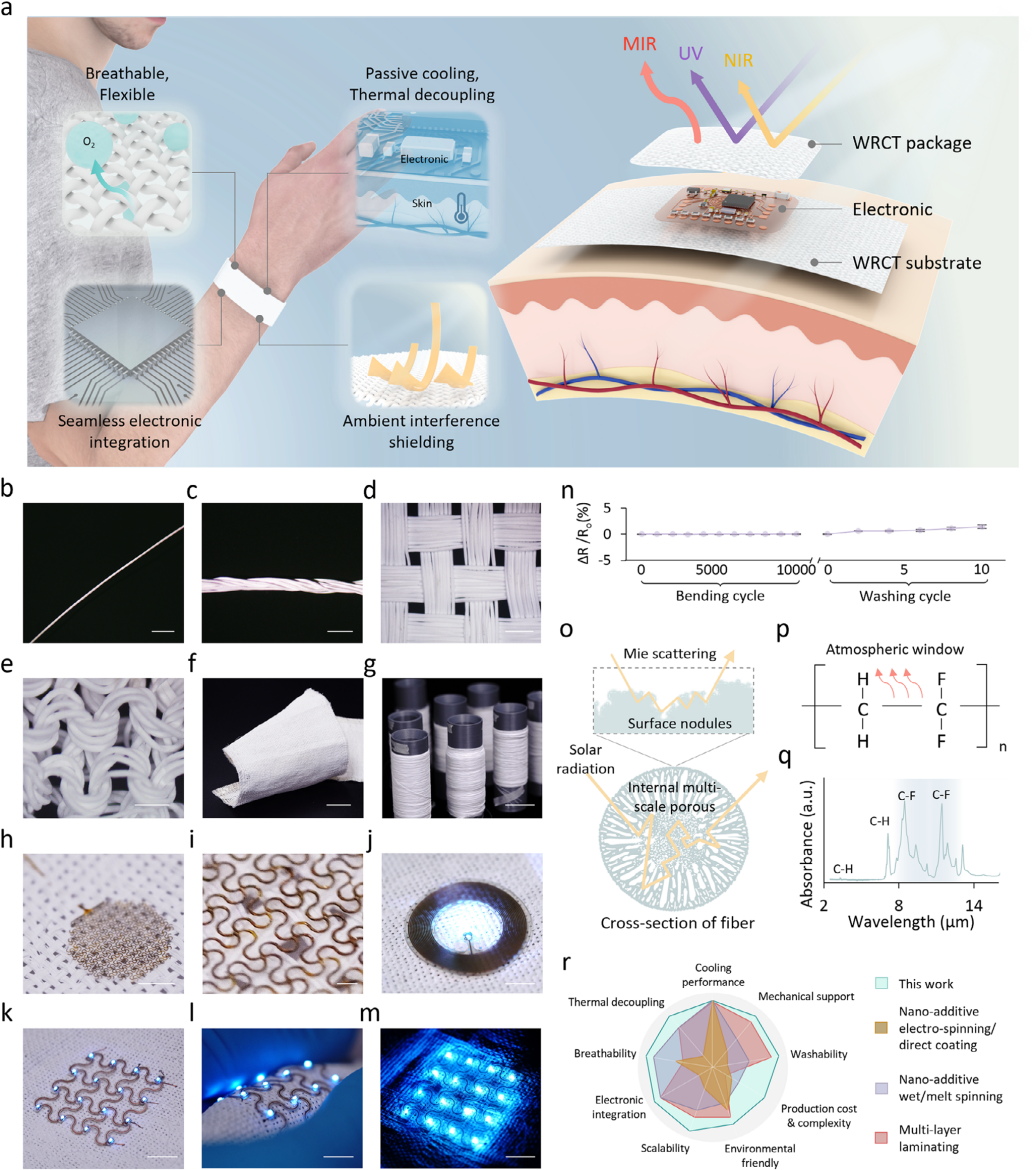
Concept, structure, scalability, and integration of WRCT and its derivatives. (a) Schematic illustration of WRCT‐based smart textiles highlighting key advantages for personal thermal management and wearable electronics. (b–e) Optical microscopy images of WRCT in different forms: single fiber (b), twisted yarn (c), woven fabric (d), and knitted fabric (e); scale bars, 2 mm (b, c), 0.5 mm (d, e). (f, g) Digital photograph of flexible and breathable WRCT woven fabric and WRCT fiber bundles with a total length exceeding 5 km produced via one‐step, additive‐free wet spinning; scale bar, 15 mm (f), 3 cm (g). (h, i) Photographs of breathable WRCT fabric electrodes seamlessly integrated with electronic components (h) and close‐up view (i); scale bar, 4 mm (h), 0.3 mm (i). (j) Wireless powering coils, integrated into the WRCT fabric, provide energy supply; scale bar, 6mm. (k–m) Photographs of a flexible LED array on WRCT fabric under different conditions; scale bars, 8 mm. (n) Electrical stability of the integrated LED array during repeated bending and washing cycles. (o) Schematic of the cauliflower‐like surface nodules and hierarchical porous structure within a single WRCT fiber formed by phase inversion kinetics, enabling strong solar reflection via Mie scattering. (p) Chemical structure of PVDF polymer that facilitates high mid‐infrared emissivity. (q) FTIR absorbance spectrum of WRCT confirming strong emission in the atmospheric transparency window (8–13 µm) via intrinsic C─F bond vibrations of PVDF. (r) Comparison of the fabrication process and performance of WRCT with conventional radiative cooling textiles.

Here, we report a wearable radiative cooling textile (WRCT) tailored for flexible electronics integration (schematic diagram shown in Figure 1a). The textile is produced from a single commodity polymer, polyvinylidene fluoride (PVDF), via technically mature, scalable wet spinning. The advantages of wet spinning enable continuous production of hundreds or even thousands of fibers in a single operation, facilitating large‐scale manufacturing [26]. These fibers are readily processed into yarns and woven or knitted into fabrics using standard textile methods (Figure 1b–g). More importantly, diverse essential electrical components (Figure 1h–m) for state‐of‐the‐art wearables can be robustly interlaced while retaining stable electrical performance after repeated bending and laundering (Figure 1f). This capability opens up unlimited possibilities for the development of advanced wearables. Specific demonstrations include flexible and permeable electrodes (Figure S1) for monitoring physiological signals (with electrocardiogram (ECG) and electromyography (EMG) monitoring shown in Figures S2 and S3), wireless powering coils for energy supply in wearable circuits, and LED arrays for human‐machine interfaces, all illustrated in Figure 1h–m.

By precisely controlling phase inversion hydrodynamics during spinning, we generate hierarchically porous microfibers with interconnected pores and cauliflower‐like surface nodules in a single, additive‐free step (Figure 1g). The resulting WRCT achieves broadband solar reflectance exceeding 95% through Mie‐resonant backscattering, mid‐infrared emissivity of 0.96 via intrinsic C─F vibrations (Figure 1p,q; Figure S4), and air‐like through‐plane thermal conductivity of 0.041 W/mK that effectively isolates electronics from the skin, while retaining the breathability and flexibility of conventional textiles. This work delivers an immediately scalable, safe, and manufacturable foundation for reliable, high‐performance, all‐day wearable electronics.

## 2 Results and Discussion

### 2.1 WRCT Design and Characterization

State‐of‐the‐art radiative‐cooling textiles achieve sub‐ambient cooling but rely exclusively on inorganic nanoparticles (e.g., TiO_2_, ZnO, SiO_2_, Al_2_O_3_) [24, 27, 28, 29, 30, 31] or complex multilayer metamaterials incorporated via electrospinning [32, 33, 34, 35], coating [36, 37], or lamination [38, 39]. These approaches inevitably sacrifice mechanical strength, elevate production costs, hinder industrial scalability, and raise environmental concerns due to particle release during wearing or laundering. We overcame these limitations by abandoning inorganic fillers entirely and exploiting the intrinsic phase inversion dynamics of a single PVDF polymer during scalable wet spinning. This additive‐free, one‐step approach simultaneously creates tailored hierarchical surface and bulk microstructures, delivering exceptional radiative cooling performance that rivals or exceeds recent benchmarks (Table S1). As summarized in the radar chart (Figure 1j), our WRCT surpasses nanoparticle‐loaded and metamaterial‐based counterparts across nearly all performance metrics, setting a new benchmark for practical, high‐performance wearable radiative cooling for outdoor applications.

**FIGURE 2:**
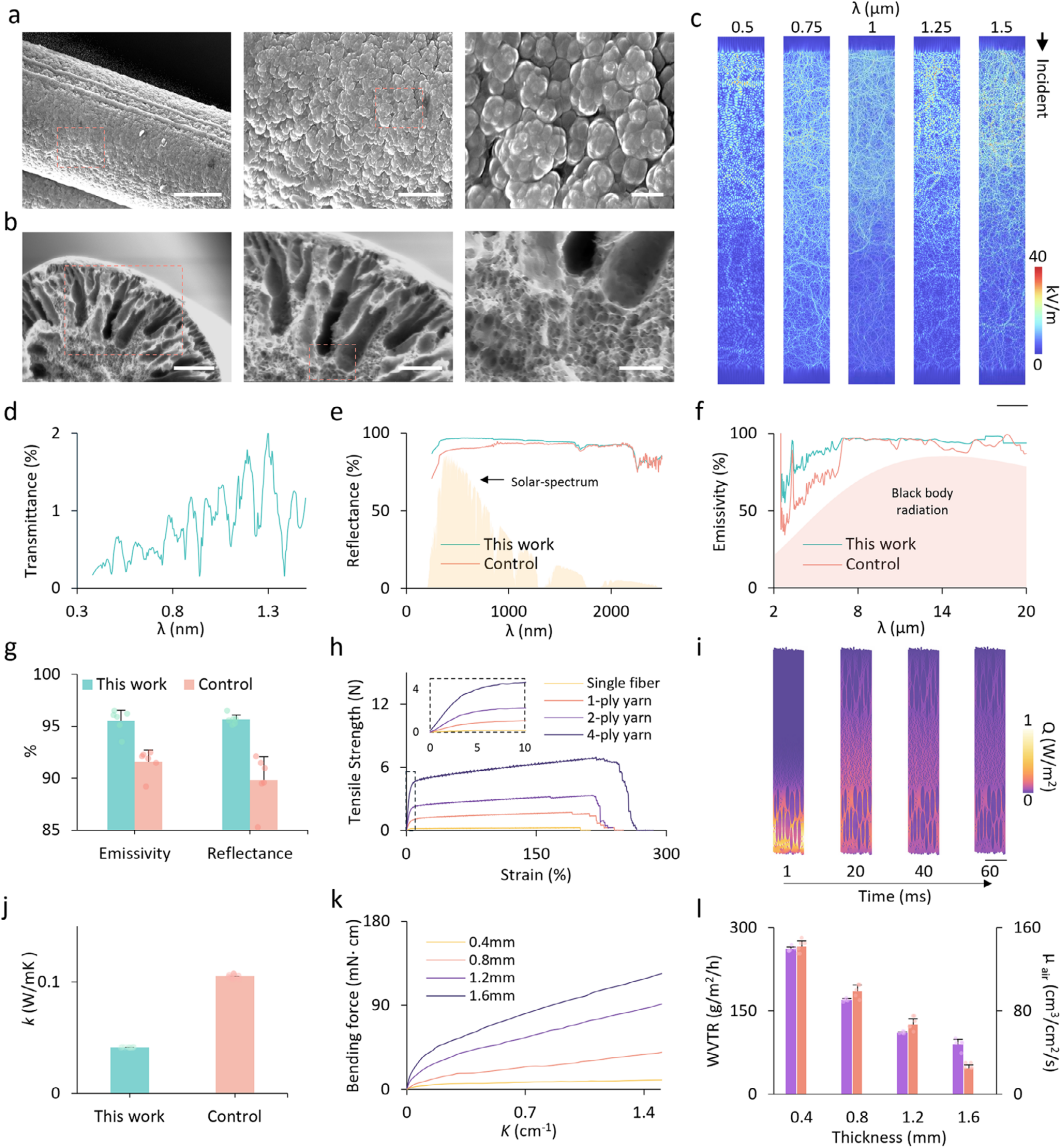
Morphology, optical, thermal, mechanical, and breathability characterization of WRCT fiber, yarn, and fabric. (a, b) SEM images of the surface (a) and cross‐section (b) of a single WRCT fiber, revealing the hierarchical porous structure formed by phase‐inversion kinetics; scale bars, 100, 30, 5 µm (a, from left to right) and 30, 20, 10 µm (b, from left to right). (c) Electromagnetic simulations of electric field distribution in the WRCT under light illumination at wavelengths of 0.5, 0.75, 1.0, 1.25, and 1.5 µm; scale bar, 10 µm. (d) Simulated transmission rate of WRCT fiber under variable wavelengths (from 0.4 to 1.5). (e–g) Solar reflectance of WRCT across 0.3–2.5 µm (e), Hemispherical infrared emissivity of WRCT across 2.5–25 µm (f), and their corresponding average values (g, mean ± s.d., *n* = 5) for WRCT and control fibers prepared by conventional wet spinning. (h) Representative tensile stress–strain curves of a single WRCT fiber and WRCT yarns with increasing ply number (1‐, 2‐, and 4‐ply). (i) Simulated heat flux distribution of Joule heating with unilateral heat source application. (j) Thermal conductivity (*k*) of WRCT fiber compared with control fiber prepared by conventional wet spinning (mean ± s.d., *n* = 10). (k) Bending force versus curvature curves of WRCT woven fabrics at different thicknesses. (l) Water vapor transmission rate (WVTR) and air permeability (μ_air)_ of WRCT woven fabrics as a function of fabric thickness (mean ± s.d., *n* = 6).

In the wet spinning process, a polymer solution is extruded through a spinneret into a nonsolvent coagulation bath, where solvent–nonsolvent counter‐diffusion induces thermodynamic instability and triggers phase inversion to form continuous fibers [40]. This diffusion exchange drives liquid–liquid phase separation, yielding a polymer‐rich phase that forms the solid matrix and a polymer‐lean phase that determines the morphology of the fiber. For WRCT fibers, PVDF is dissolved in N,N‐dimethylacetamide (DMAc) and extruded into a saturated aqueous NaCl coagulation bath. Rapid counter‐diffusion of solvent and nonsolvent triggers instantaneous NaCl supersaturation at the interface, precipitating microcrystals that act as transient templates (phase inversion schematic in Figure S5) [41, 42]. These templates drive PVDF chain collapse and micelle aggregation, forming cauliflower‐like nodular protrusions (0.8–1.6 µm diameter) on the fiber surface (Figure 2a,b). Beneath the nascent skin, the newly formed polymer layer functions as a semi‐permeable membrane. Osmotic pressure sustains DMAc outflow, inducing Rayleigh‐Taylor instability [43] and finger‐like macrovoids [44] (major axis 10–50 µm, minor axis 3–8 µm) that extend 30–50 µm inward. As solvent depletion progresses toward the core, diffusion slows, transitioning the solidification mechanism from hydrodynamic fingering to spinodal decomposition and yielding a bicontinuous sponge‐like network (pore size 0.4–0.8 µm). Unlike conventional wet spinning, which employs high draw ratios to densify fibers by collapsing pores, aligning polymer chains, and reducing void volume [45], we deliberately minimized applied stress (low drawing ratio) to preserve the as‐formed surface nodules and internal porosity essential for efficient Mie‐resonant scattering and radiative cooling performance.

To quantitatively demonstrate the contribution of surface nodules and multiscale porosity to solar reflectance, we conducted finite‐element method (FEM) simulations using COMSOL Multiphysics (Wave Optics Module). The 2D fiber model was reconstructed from SEM cross‐sections, incorporating the observed surface nodules and hierarchical internal porosity. Incident solar radiation (wavelength range 0.3–2.5 µm, encompassing the primary solar spectrum for daytime radiative cooling) was applied to the model. The simulated electric field distributions (Figure 2c) reveal that the synergistic combination of surface nodules and multiscale porosity induces strong Mie‐resonant backscattering across the solar spectrum. Incident light undergoes repeated resonant scattering and attenuation within the fiber, yielding excellent solar reflectance and negligible transmission (Figure 2d).

To validate the critical role of this hierarchical architecture, we fabricated PVDF fibers using the widely adopted conventional wet spinning method (higher polymer content, pure water coagulation bath, high draw ratio) [46, 47, 48], yielding smooth surfaces and denser cores as the control (Figure S6). The optimized WRCT fiber exhibits 95.6% average solar reflectance (300–2500 nm) versus 89.8% for the control (Figure 2e–g). Dual‐scale scattering synergy: surface nodules (0.8–1.6 µm) induce Mie scattering for visible to short‐wave near‐infrared (400–1200 nm), while interconnected internal pores (0.4–0.8 µm) enhance Rayleigh‐Mie hybrid scattering for mid‐to‐long wave near‐infrared (1200–2500 nm), collectively achieving broadband solar reflectance >95%. In the mid‐infrared region of the electromagnetic spectrum, the WRCT achieves an overall infrared emittance of 0.96 (versus 0.89 for control). This enhancement arises from the hierarchically porous structure formed via phase inversion, which combines an exceptionally high internal surface area with sub‐micron polymer wall thicknesses, substantially increasing the volume density of radiating C─F bond dipoles while retaining the strong intrinsic infrared absorption of PVDF.

With an average diameter of ∼220 µm, single WRCT fibers sustain ∼250 mN breaking force, sufficient for embedding flexible electronics without reinforcement. The fibers remain fully compatible with conventional textile processing: twisting, plying, and weaving readily tailor mechanical performance. For example, a 2‐ply, 16‐filament yarn (∼50 turns/m) achieves 3.2 N breaking strength (Figure 2h; Figure S7). In this work, fibers were woven into the fabric shown in Figure 1b (right) for subsequent device integration.

The resulting WRCT fabric exhibits ultralow thermal conductivity (∼0.041 W/mK compared with ∼0.10 W/mK for the dense control) owing to extensive air entrapment and highly tortuous heat‐transfer paths within the bicontinuous pore network (Figure 2i,j; Video S1). The hierarchical porosity not only suppresses thermal conduction but also imparts low bending rigidity (<10 mN·cm at a curvature of 1.4 cm^−1^ for 0.4 mm thickness of fabric), as the presence of macrovoids and submicron pores reduces effective material stiffness and facilitates easier deformation. This low bending rigidity, combined with high air permeability (142 cm^3^/cm^2^/s) and substantial water vapor transmission rate (261 g/m^2^/h), ensures the fabric retains excellent breathability, wearability, and overall comfort comparable to conventional textiles (Figure 2k,l), making it highly suitable for next‐generation wearable electronics and passive radiative cooling applications. Ongoing and future efforts to reduce fiber diameter (enhancing softness and wearing comfort while preserving radiative cooling performance) and to integrate multi‐layer sweat‐management designs (e.g., directional liquid transport layers) are expected to eliminate sweat‐related effects on radiative cooling and expand WRCT's applicability across diverse textile domains. While the WRCT fabrication process is per‐ and polyfluoroalkyl substances (PFAS) free, potential trace residuals from upstream polymer production and the long‐term environmental persistence of fluoropolymers warrant continued attention. Future adoption of PFAS‐free or sustainably synthesized PVDF alternatives could further minimize any lifecycle‐related environmental impact.

### 2.2 Passive Radiative Cooling Performance of WRCT Fabric

**FIGURE 3:**
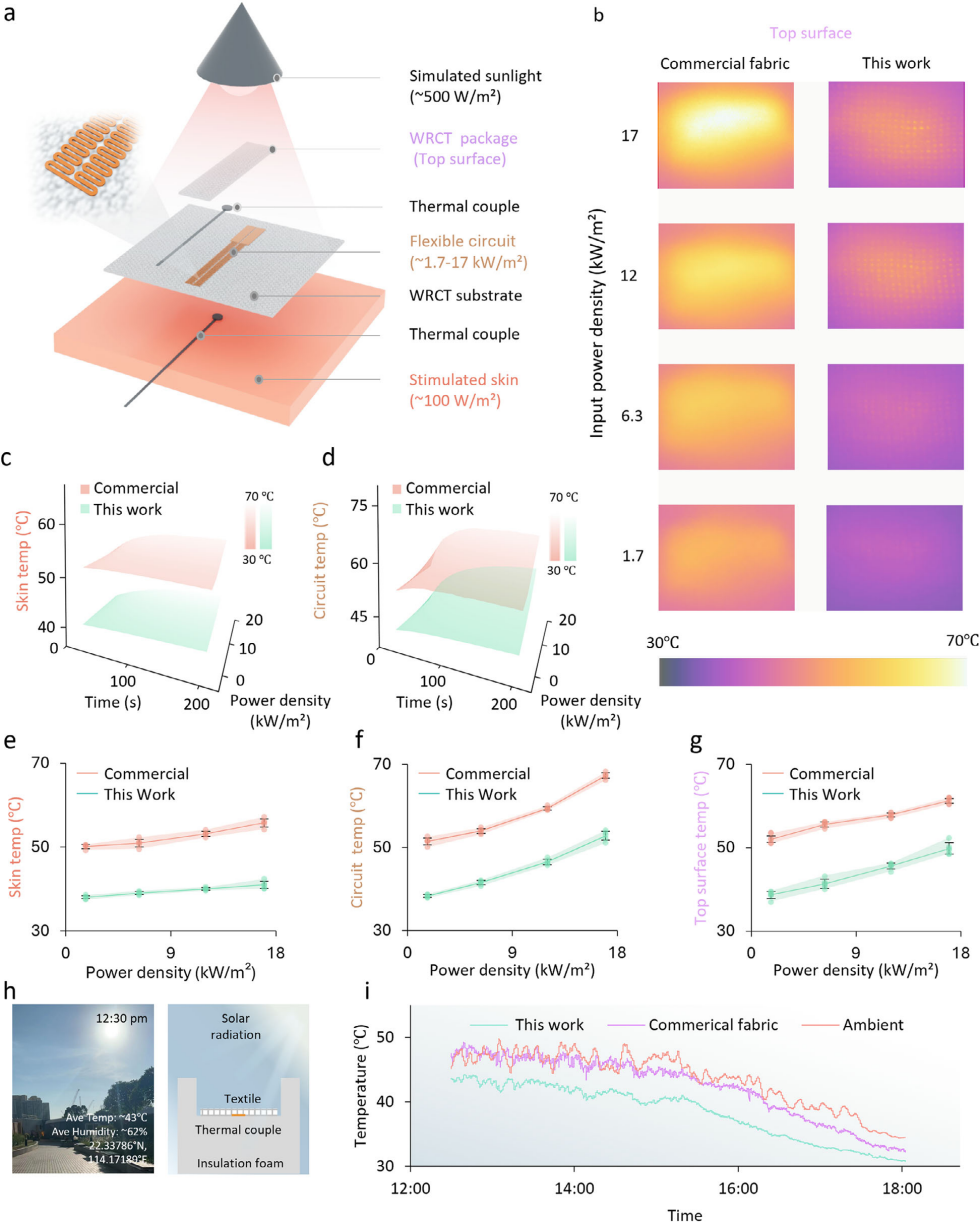
Passive radiative cooling performance of WRCT fabric. (a) Schematic of the indoor experimental setup mimicking on‐body wearable conditions, consisting of a solar simulator (0.5 Sun), a flexible Cu/PI serpentine heater sandwiched between WRCT woven fabric and a simulated skin layer. (b) Thermal infrared images comparing the top‐surface temperature of WRCT and commercial apparel fabric packages at input power densities ranging from 1.7 to 17 kW/m^2^. (c, d) Time‐dependent temperature profiles of the simulated skin (c) and the flexible Cu/PI serpentine circuit (d) under different applied currents. (e–g) Steady‐state temperatures of the simulated skin (e), flexible serpentine circuit (f), and top surface of the fabric package (g) as a function of applied power density, mean ± s.d., *n* = 5. (h) Photograph of the outdoor measurement setup (right) and the experiment setup (left). (i) Continuous temperature recordings from 12:30 to 18:00 on July 16, 2025, demonstrate the cooling performance of WRCT fabric compared to commercial apparel fabric under real sunlight conditions.

To quantify the real‐world passive cooling capability of the WRCT fabric when integrated with flexible electronics, we constructed the outdoor‐mimicking test platform shown in Figure 3a. A serpentine Cu/PI flexible circuit (area: ∼142 mm^2^, detailed dimensions and simulation are provided in Figures S8 and S9) served as a programmable Joule heat source to replicate the thermal load of typical wearable devices. The flexible circuit was sandwiched between two layers of WRCT fabric to maximize solar rejection and promote radiative heat loss while maintaining intimate contact with a silicone‐based simulated skin.

The simulated skin was supplied with a constant basal heat flux of ∼100 W/m^2^ (matching human metabolic heat dissipation in outdoor environments [36]), and the entire assembly was exposed to 500 W/m^2^ of simulated AM1.5G sunlight (0.5 Sun). Different constant current levels (0.1–0.4A) were applied to the serpentine heater, corresponding to additional localized power densities of ∼1.7to∼17 kW/m^2^. Temperatures of the flexible circuit and the simulated skin surface were continuously monitored by calibrated thermocouples, and thermal images of the top fabric surface were recorded using an infrared camera after thermal equilibrium was reached (Figure 3b). For direct comparison, an identical test was performed by replacing the WRCT with a commercially available polyester apparel fabric (152 g/m^2^, widely used in summer sportswear), illustrated in Figure S10.

Figure 3c,d and Figure S11 present the time‐dependent temperature rise of the flexible circuit and simulated skin at different applied currents. Figure 3e–g summarizes the equilibrium temperatures of key components (circuit, simulated skin, and top surface). Thermal imaging reveals the superior solar reflectance and radiative cooling performance of WRCT, with the top surface of the WRCT assembly remaining up to 13.4°C cooler than that of the control under identical conditions (Figure 3b–d). Also, the temperature of the sandwiched flexible circuit is reduced by 12.5°C–14.5°C compared to the control. The lower operating temperature delivers an additional key advantage for wearable electronics. Lower operating temperature reduces the electrical resistance of Cu traces (positive temperature coefficient of Cu), requiring less voltage to sustain constant current and thereby decreasing Joule heat generation and ultimately extending device lifespan. Thus, WRCT simultaneously provides powerful passive cooling and enables more energy‐efficient operation of integrated flexible devices under real‐world solar exposure.

The most critical safety advantage of WRCT is its ability to keep skin temperature in a safe and comfortable range (Figure 3c,e). At the highest heat load (0.4 A, corresponding to ∼17 kW/m^2^ device power over a 142 mm^2^ area under 0.5 Sun illumination), simulated skin temperature decreases from 55.8°C with commercial polyester to only 40.9°C with WRCT, representing a remarkable 14.9°C reduction that maintains skin well below the thermal discomfort threshold (∼43°C). The highly porous structure of WRCT effectively decouples device‐generated heat from the skin, directing most of the heat toward the top WRCT layer, where it is subsequently dissipated through emission and convection.

We further performed outdoor experiments to assess the dynamic passive cooling performance of WRCT under real‐world conditions (Figure 3h). The WRCT and control commercial fabric were placed on insulating foam, with a thermocouple positioned at the fabric‐foam interface to continuously monitor temperature from 12:30 to 18:00 in Hong Kong (22.33786°N, 114.17189°E). During peak solar irradiation, the WRCT surface stayed 5.2°C–5.5°C cooler than the polyester control and consistently 7.2°C–7.8°C below ambient air temperature (Figure 3i). This result directly confirms the robust passive radiative cooling capability of WRCT in actual outdoor environments.

### 2.3 WRCT‐Integrated Wearable Devices

**FIGURE 4:**
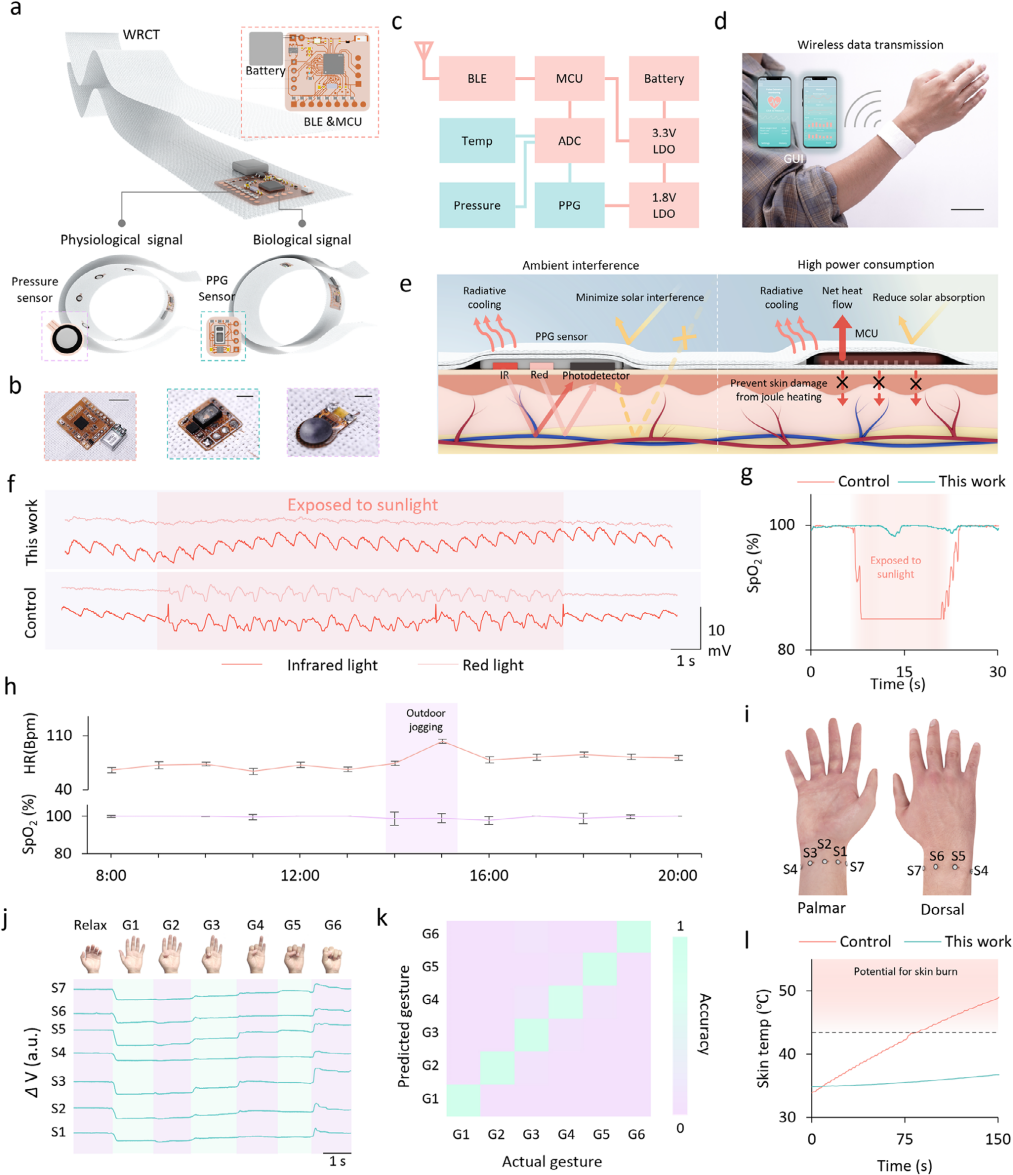
Multifunctional WRCT‐based smart wristband for healthcare monitoring, gesture recognition, and thermal management. (a) Schematic of the textile‐integrated multifunctional wristband capable of simultaneous physiological (contact pressure) and biological (PPG) signal acquisition for continuous health monitoring and hand‐gesture recognition. (b) Photographs of the key electronic components: MCU and BLE module (left), PPG sensor (middle), and pressure sensor (right); scale bar, 10, 2, 3 mm (from left to right). (c) Block diagram showing the circuit architecture and data‐flow logic of the wristband system. (d) Photograph of the fully integrated WRCT wristband worn on the wrist (left) and the custom smartphone GUI displaying real‐time wirelessly transmitted data (right); scale bar, 5 cm. (e) Schematic illustrating the advantages of WRCT for outdoor wearable applications: mitigation of solar optical interference on PPG sensors (left) and thermal management and decoupling to prevent skin burns from high‐power electronics (right). (f) Real‐time PPG waveforms acquired by the WRCT wristband (top) and a control wristband made of commercial apparel fabric (bottom) under simulated sunlight irradiation (500 W/m^2^). (g) Calculated blood oxygen saturation (SpO_2_) values from the WRCT and control wristbands under the same simulated sunlight exposure (500 W/m^2^). (h) Continuously monitored heart rate and SpO_2_ levels throughout the day (8:00–20:00) using the WRCT wristband, mean ± s.d., *n* = 60. (i) Palmar and dorsal views of the hand diagram showing the placement locations of pressure sensors for hand‐gesture detection. (j) Voltage signals from multiple pressure sensors during various hand gestures. (k) Confusion matrix (heatmap) showing prediction accuracy of hand gestures using a self‐developed neural network algorithm, achieving ∼93% overall accuracy for the total 6 hand gestures. (l) Temperature rise of the simulated skin beneath the wristband during continuous gesture‐recognition processing and wireless data transmission under simulated sunlight irradiation (500 W/m^2^), comparing WRCT with commercial apparel control fabric (control).

To demonstrate the practical advantages and seamless integration capability of WRCT for flexible electronics, we developed a textile‐integrated multifunctional smart wristband designed for long‐term wear and outdoor use (Figure 4a). The device supports different types of sensors, including a photoplethysmography (PPG) sensor (Figure S12) for real‐time monitoring of heart rate, pulse waveform, and blood oxygen saturation (SpO_2_), as well as a pressure sensor array distributed across the wrist cross‐section to enable gesture recognition. These functions support potential applications in continuous health monitoring, rehabilitation assessment, and human‐computer interaction, including virtual reality interfaces.

A flexible printed circuit board integrating a microcontroller (MCU) and Bluetooth Low Energy (BLE) module was directly laminated onto the WRCT fabric and fully encapsulated with an additional WRCT layer, providing mechanical protection, breathability, and effective thermal management (Figure 4b). Interconnection cables were effortlessly routed through the open voids of the woven structure, allowing seamless electrical bridging between components on opposite fabric surfaces without requiring rigid substrates or complex wiring. The system architecture and key components are shown in the block diagram in Figure 4c. Sensor signals are digitized and processed by the onboard MCU, then wirelessly transmitted via BLE to a smartphone, where data are visualized in real time through a custom graphical user interface (Figure 4d; Figure S13). Figure 4e schematically illustrates the multifunctional capabilities and benefits enabled by WRCT across different application scenarios.

For sensors susceptible to ambient interference, such as the PPG sensor that relies on the emission, transmission, and detection of optical signals, solar irradiance can severely degrade the signal‐to‐noise ratio and induce large measurement errors in outdoor environments. Figure 4f compares the acquired PPG pulse signals under simulated solar irradiation (500 W/m^2^) for the WRCT‐integrated device versus a control device (using the same commercially available apparel fabric from previous tests). By reflecting nearly all incident solar radiation, WRCT sharply mitigates optical interference, thereby markedly improving the stability and accuracy of pulse waveform and SpO_2_ monitoring under direct sunlight (Figure 4g).

Across all operating conditions, WRCT continuously cools the underlying skin and electronics through strong sub‐ambient radiative cooling and maintains long‐term wearing comfort by providing excellent air permeability and water vapor transmission, effectively preventing moisture buildup and thermal discomfort, allowing all‐day wearing without observed discomfort or skin irritation (Figure 4h).

In another demonstration, we employed seven pressure sensors across the wrist cross‐section for hand gesture recognition. The sensor positions, corresponding signals under various hand gestures, and prediction accuracy are shown in Figure 4i–k. During computationally intensive real‐time gesture recognition processed using a neural network, which requires high sampling rates, complex algorithms, and simultaneous wireless transmission, the MCU generates significant self‐heating. WRCT not only blocks solar absorption and dissipates device heat via powerful radiative cooling, but its ultralow thermal conductivity also forms a robust thermal barrier that prevents heat from reaching the skin, substantially reducing burn risk (Figure 4l). Notably, the control device rapidly exceeded 43°C (the skin burn threshold) after approximately 75 s of operation, whereas the WRCT‐integrated system exhibited a much slower temperature rise, ensuring prolonged safety and usability.

## 3 Conclusion

This study reconciles the conflicting thermal‐optical demands of wearable electronics and human physiological comfort/safety through the development of a monolithic, additive‐free WRCT. Unlike conventional strategies that rely on fragile inorganic nanoparticle fillers or complex multilayer laminates, our approach exploits the intrinsic hydrodynamics of phase inversion to transform a single commodity polymer (PVDF) into a hierarchical architecture. By strictly controlling solvent counter‐diffusion, we achieved a fiber morphology characterized by Mie‐resonant surface nodules and a multi‐scale porous core, delivering an ultra‐high solar reflectance (>95%) and mid‐infrared emissivity (0.96).

This architecture provides a unique triple‐mode solution to the conflicting thermal‐optical requirements of wearable systems. First, its near‐perfect broadband reflection creates an optical shield that suppresses photothermal noise, ensuring the signal fidelity of optoelectronic sensors (such as PPG) even under direct sunlight. Second, the material's air‐like thermal conductivity (0.041 W/mK) effectively thermally decouples power‐dissipating electronics from the skin. We demonstrated that this insulation maintains skin temperatures safely below the low‐temperature burn threshold (∼43°C), even when subjecting the system to intense solar irradiation (∼500 W/m^2^) and localized device heating loads (∼17 kW/m^2^) that can cause rapid injury with conventional clothing textiles. Finally, the porous structure of fabric preserves the breathability and mechanical compliance essential for long‐term physiological comfort.

Critically, this performance is achieved using the globally established wet spinning process, ensuring immediate industrial scalability and compatibility with standard textile weaving and knitting infrastructure. By eliminating the reliance on nanomaterials, the WRCT offers a mechanically robust, environmentally benign, and cost‐effective platform. Consequently, this work establishes the WRCT not merely as a novel cooling material, but as a practical, manufacturable platform for safe, high‐performance, all‐day wearable electronic interfaces.

## 4 Methods

### 4.1 Fabrication of WRCT

PVDF powder (Kynar 761, Arkema) was dried in a vacuum oven at 80°C for 12 h to remove adsorbed moisture. A 10 wt.% spinning dope was prepared by dissolving the dried PVDF powder in DMAc (99.8%, Mackin) with ultrasonication at 60°C for 2 h until a homogeneous and transparent solution was obtained (Figure S14). The resulting dope was filtered through a 100 µm stainless‐steel mesh to remove any undissolved particles or impurities, and finally transferred into a syringe equipped with an 8‐hole spinneret (each hole: ∼260 µm inner diameter).

Wet spinning was performed using a multi‐filament spinning system (Figure S15). The dope was extruded simultaneously through the 8‐hole spinneret at a controlled air pressure of ∼1.5 MPa using an air compressor pump. The extruded dope streams entered a coagulation bath containing saturated aqueous NaCl solution (≈36 wt.%) at room temperature. Phase inversion occurred immediately upon contact with the non‐solvent, forming solid fibers. The nascent fibers were drawn through the bath (bath distance: 100 cm) at a minimum stress and continuously taken up by a motorized winder at a linear speed of ∼3.5 m/min. The as‐spun fiber bundle was collected onto a 3D‐printed cone (diameter: 33mm).

To fully remove residual NaCl and DMAc, the collected fibers were immersed in flowing deionized water for 48 h with frequent water changes. The purified fibers were air‐dried under ambient conditions for 24 h and subsequently vacuum‐dried at 60°C for 2 h. For comparison, a control fiber highlighting the benefits of the hierarchical structure was prepared under modified conditions: 20 wt.% PVDF/DMAc dope, deionized water as coagulation bath, and 50% draw ratio applied at the winder.

The resulting WRCT fibers were then twisted into yarns or directly woven into fabrics using a standard weaving loom (Figure S16). The specification of the woven fabric is listed in Table S2.

### 4.2 Fabrication and Demonstration of Permeable WRCT Electrode

A polydimethylsiloxane (PDMS) layer was spin‐coated (500 rpm, 30 s) onto a temporary glass substrate and cured at 100°C for 10 min. A 20‐µm‐thick polyimide (PI) film was then laminated onto the cured PDMS and patterned into an open‐mesh structure (50 µm trace width) using laser ablation (LPKF ProtoLaser U4). The resulting open‐mesh PI scaffold was transferred using water soluble tape. Subsequently, Ti/Au (5/50 nm) was deposited onto the patterned PI film by electron‐beam evaporation. Finally, the water soluble tape was dissolved in deionized water, yielding a free‐standing, self‐supported open‐mesh electrode. The electrodes were laminated on WRCT by using silicone adhesive (K‐705, Kafuter, China). The detailed geometrical specifications can be found in our previous article [5]. The permeable WRCT electrodes were used to acquire electrocardiogram (ECG) and electromyogram (EMG) signals from a human volunteer. Data were recorded using a PowerLab 16/35 system equipped with a Bio Amp front‐end (PL3516, AD Instruments, Australia).

### 4.3 Characterization of WRCT

The FTIR spectra of the PVDF powder were recorded using the PerkinElmer Spectrum II FT–IR Spectrometer. The cross‐section morphology of WRCT fibers was examined using an FEI Quanta 450 FEG environmental scanning electron microscope (SEM) operated at 10 kV. Samples were fractured by immersion in liquid nitrogen for 30 s, followed by sputter‐coating with a ∼10 nm gold layer.

Spectral solar reflectance and infrared emissivity of WRCT fabrics were measured using a PerkinElmer Lambda 1050+ UV/VIS/NIR spectrophotometer equipped with an integrating sphere and a Bruker Vertex 70 FTIR spectrometer, respectively, at a sample thickness of ∼1.2 mm. Tensile properties of WRCT fibers/yarns were evaluated using an Instron 5942 Micro Tensile Tester in accordance with ASTM D3822. Air permeability of WRCT fabrics was measured using an SDL Atlas MO21A Electronic Air Permeability Tester at a pressure difference of 25 Pa. Water vapor transmission rate of WRCT fabrics was determined following ASTM E96. Bending properties of WRCT fabrics were evaluated using a KES‐FB2‐S Pure Bending Tester (Kato Tech Co., Japan).

The thermal conductivity (*k*) of WRCT fabrics was evaluated using a TCi thermal conductivity analyzer (C‐Therm, Canada), as per ASTM D5930‐17. The all‐dimensional characteristics were quantitatively determined using ImageJ (National Institute of Health, USA). Experiments on WRCT fabrics of different thicknesses were conducted by stacking multiple layers of the same fabric.

### 4.4 Passive Cooling Performance of WRCT

The overall experimental setup is illustrated in Figure 3a. The experiment was conducted in an indoor environment (Temperature ∼21°C, Relative humidity ∼60%). A flexible circuit, serving as a simulated heat source mimicking electronic components in a wearable system, was fabricated by laser cutting (LPKF ProtoLaser U4) a Cu/PI thin film. The film was laminated onto the bottom substrate using silicone adhesive (K‐705, Kafuter, China) and onto the top layer using thermally conductive silicone adhesive (K‐5205, Kafuter, China). Simulated solar illumination was provided by a xenon lamp system (CEL‐HXF300‐T3, CEAuLight, China), with light intensity calibrated using a radiometer (CEL‐FZ‐A, CEAuLight, China). Temperature measurements were performed using ultrafine (0.05 mm) T‐type thermocouples (TT‐T‐44, Kaipusen, China) to minimize geometric interference with the cooling performance. Thermal images were acquired with a FLIR A615 infrared camera. The outdoor experiment was performed at 22.33786°N, 114.17189°E, Hong Kong, from 12:30 to 18:00 on July 16, 2025. The thermocouples used are the same as the above‐mentioned ones. The WRCT and control samples, each approximately 0.8 mm thick, were placed in an outdoor environment for 30 min prior to data acquisition.

### 4.5 Electrical Hardware

The circuit board tailored for the WRCT wearable wristband is a compact, battery‐powered Bluetooth Low‐Energy (BLE) biometric module with a well‐defined power domain that utilizes VBAT (2.5–4.2 V, such as a Li‐ion cell) to feed two LDOs (TPS76933 providing 3.3 V, 100 mA for digital and radio rails, and TLV75718 supplying 1.8 V, 1 A for the MAX30102 optical sensor) (Figure S17). Its digital core is centered around the nRF52832 BLE SoC (Cortex‐M4, 2.4 GHz transceiver), clocked by a 32 MHz crystal (X2) for the radio and CPU, and a 32.768 kHz crystal (X3) for the RTC/low‐power sleep. The human‐interface/sensing section features the MAX30102 integrated pulse oximeter and heart‐rate sensor (I^2^C slave). The radio front‐end incorporates a 2.4 GHz chip antenna (2450AT18A100E) with a π‐matching network for a 50 Ω match. For analog expansion, a 9‐pin header exposes eight Analog‐to‐digital converter (ADC) inputs (AIN0–AIN7) to external pressure sensors, with each line featuring a 10 kΩ pull‐up to VDD for biasing or resistive sensor excitation. Overall, the board functions as a compact, battery‐powered BLE biometric module that measures heart rate and SpO_2_ via the MAX30102, processes data on the nRF52832, and transmits results over BLE, while also offering eight auxiliary analog channels for pressure sensors (RF‐C7.6‐L11‐LF, Runeskee, China), for the hand gestures prediction function demonstrated in this study.

### 4.6 Fabrication and Demonstration of WRCT Wearable Wristband

The fabrication of the WRCT wearable wristband is based on the pattern illustrated in Figure S18. A total of two layers of WRCT fabric were used as the substrate and package. The lamination of PPG/ pressure sensors and Microcontroller Unit (MCU) was used in the same way as the experiment for the passive cooling performance sample. Velcro was used for connecting the two ends of the bands and for tightness adjustment on the wrist. The fabric edges were folded and joined together with adhesive interlining. A control device was made by substituting WRCT with the commercially available polyester apparel fabric used in the passive cooling performance evaluation experiment. To demonstrate the WRCT wristbands connected with the PPG sensor, a volunteer wore the wristband, and simulated sunlight (500 W/m^2^) was temporarily applied on the device to evaluate the effect of solar irradiation on the PPG signal. The WRCT wristbands were also worn by a volunteer for 12 h, while the PPG signal was acquired every hour. For the WRCT wristband connected to pressure sensors, signal acquisition is performed using the multi‐channel ADC integrated into the MCU, with the sampling rate of each channel configured to 100 Hz. Constrained by the limited computing performance of the MCU, we implemented a streamlined three‐layer Convolutional Neural Network (CNN) model within the embedded program to perform the gesture recognition task on the pressure signals collected by the wristband. The three‐layer CNN is tailored to the MCU's limited computing resources: it consists of a 1D convolutional layer (8 filters, 1 × 3 kernel) for extracting temporal features from the pressure signals, a max pooling layer for feature downsampling, and a fully connected layer for final gesture classification.

### 4.7 Finite Element Analysis

#### 4.7.1 Optical Simulation

The optical performance of the WRCT fiber was investigated using the Wave Optics Module in COMSOL Multiphysics. To accurately replicate the hierarchical porous morphology observed in SEM images and our structural design, a customized Python script was developed to generate the 2D geometry, which was subsequently imported into COMSOL via a subroutine. The porous architecture was constructed using non‐overlapping circles and ellipses of varying dimensions, with an algorithm ensuring that the geometric distribution statistically matched the experimental microscopic observations. Material properties for PVDF and air were adopted directly from the standard material database. Boundary conditions were established following the configuration for periodic microstructures: periodic boundary conditions were applied to the lateral boundaries to eliminate the influence of material boundary scattering in the transverse direction, while the incident light was launched from the input port. Perfect matching layers were employed at both the input and output ends to absorb outgoing waves and suppress spurious boundary reflections. Post‐processing of the simulation data quantified the transmittance of a single fiber across different wavelengths of the solar spectrum.

#### 4.7.2 Thermal Simulation

A transient heat transfer simulation was conducted to visualize the temporal evolution of heat flux distribution within the fabric‐skin interface. The geometric model consisted of WRCT fiber in direct contact with a skin layer. Thermal boundary conditions were defined to mimic physiological conditions: the bottom surface of the fabric was maintained at a constant temperature of 37°C, while the top surface was assigned a heat flux boundary condition to represent heat dissipation into the environment. All other boundaries were set as adiabatic. The ambient temperature was fixed at 20°C. The resulting heat flux distribution maps at different time steps were analyzed to demonstrate the thermal insulation capability of the WRCT at the mesoscopic scale.

#### 4.7.3 Mechanical Simulation

The 3D models of the WRCT electrode and flexible Cu/Pi serpentine wire were created in Rhino 7 (Robert McNeel & Associates, USA) based on the dimensions of the experimental samples and then meshed in MSC Apex 2020. The meshed models were imported into Marc Mentat 2020 for FEM construction. Friction was neglected in the finite element analysis. The mechanical properties for simulation were based on the experimental data.

### 4.8 Statistical Analysis

All quantitative data were expressed as mean ± standard deviation (SD).

## Conflicts of Interest

The authors declare no conflicts of interest.

## Supporting information




**Supporting File**: advs74872‐sup‐0001‐SuppMat.docx.

**Supporting File**: advs74872‐sup‐0002‐VideoS1.mp4.

## Data Availability

The data that support the findings of this study are available on request from the corresponding author. The data are not publicly available due to privacy or ethical restrictions.
