# Supplementary material for: Wearable Tailored Passive Radiative Cooling Textile for Flexible Electronic Integration
Source: Adv Sci (Weinh). 2026 Mar 18;13(31):e24380. doi: 10.1002/advs.202524380 (PMC13587978; doi:10.1002/advs.202524380)
Supplement: Supplementary file 1 [file ADVS-13-e24380-s002.docx]

**Wearable Tailored Passive Radiative Cooling Textile for Flexible Electronic Integration**

Lung Chow^1^**^†^**, Jianpeng Zhang^1,3^**^†^**, Zehua Peng ^2^**^†^**, Denglin Ye^1^**^†^**, Binbin Zhang^1,3,4^, Jian Li^1^, Wooyoung Park^1^, Chentao Du^1^, Xingcan Huang^1^, Chun Ki Yiu^1,3^, Yuze Qiu^1^, Jingkun Zhou^1,3^, Zhenlin Chen^1^, Yuyu Gao^1^, Weibin Zhu^1,3^, Pengcheng Wu^1^, Guangyao Zhao^1^, Qiang Zhang^1,5^, Yuan Guo^1^**,** Zuowei Sun^1^, Zhan Shen^1^, Shuaiqi Ren^1^, Sang Min Won^6*^, Jiyu Li^2*^, Xinge Yu^1,3,5*^

^1^ Department of Biomedical Engineering, City University of Hong Kong, Hong Kong 999077, China

^2^ School of Biomedical Engineering, Sun Yat-sen University, Shenzhen, China

^3^ Hong Kong Centre for Cerebro-Cardiovascular Health Engineering, Hong Kong 999077, China

^4^ Key Laboratory of Photonic-Electronic Integration and Communication-Sensing Convergence, School of Information Science and Technology, Southwest Jiaotong University, 611756, Chengdu, China

^5^ Institute of Digital Medicine, City University of Hong Kong, Kowloon, Hong Kong, China

^6^ Department of Electrical and Computer Engineering, Sungkyunkwan University (SKKU), Suwon 16419, Republic of Korea

^†^These authors contributed equally to this work.

*Corresponding author, Email: [sangminwon@skku.edu](mailto:sangminwon@skku.edu); [lijy887@mail.sysu.edu.cn](mailto:lijy887@mail.sysu.edu.cn); [xingeyu@cityu.edu.hk](mailto:xingeyu@cityu.edu.hk)

**Keywords**: Wearable electronics, Textile electronics, Radiative cooling, Thermal-Optical Regulation

**This file includes:**

Figures S1 to S18

Table S1

Legends for Video S1

Figure S1. Simulated result of strain distribution of the developed breathable electrode under bending and twisting by finite element methods, demonstrating the exceptional flexibility and conformity that compatible with electronic integration.

Figure S3. Electrocardiogram (ECG) signal obtained by using the developed breathable electrode (Fig.1h & i)

Figure S4. Electromyography (EMG) signal obtained by using the developed breathable electrode (Fig.1h & i)

Figure S2. Energy-dispersive X-ray spectroscopy (EDS) image demonstrating elemental distribution of carbon (C) and fluorine (F).

Figure S5. Schematic diagram of the phase inversion process of the WRCT fiber, creating the hierarchical structure with internal multi-scale porous and surface nodules.

Figure S6. Scanning electron microscope (SEM) images of the control PVDF fiber for comparison with WRCT fiber, scale bar: 100μm (Top), 20μm (Bottom).

Figure S7. The relationship between the tensile strength and thickness of WRCT fiber and yarn at different plying number.

Figure S8. The geometric parameters and digital photo of the flexible Cu/Pi serpentine wire as the heat source for simulating the heat generation of electronics in the wearable system. a, Overall dimension of the wire. b, Detailed dimension of the serpentine structure. c, Cross sectional view of the Cu/Pi thin film showing the thickness of each layer. d, Digital photo of the Cu/Pi serpentine wire laminated on the WRCT fabric.

Figure S9. Simulated result of strain distribution of the developed flexible Cu/Pi serpentine wire under bending and twisting by finite element methods.

Figure S10. Demonstration and details of the commercial apparel polyester fabric which is 0.46mm in thickness and 152g/m^2^ in fabric weight. a, Digital photo of the commercial apparel polyester fabric, scale bar, 3cm. b, Microscopic view of the commercial apparel polyester fabric, scale bar, 1mm. c, SEM image of the commercial apparel polyester fabric, scale bar: 400 μm.

Figure S11. The temperature rise of the simulated skin (right) and the flexible Cu/PI serpentine wire (left) under different applied current and under 0.5 sun solar irradiance.

Figure S12. Digital photo of the photoplethysmography (PPG) sensor on human finger, scale bar, 5mm.

Figure S13. Graphical user interface (GUI) design for displaying real-time wirelessly transmitted data from the smart WRCT wrist band. a, Home page of the GUI showing the real-time pulse wave, blood oxygen level, heart rate and predicted body condition. b, History page which illustrates the previous blood oxygen level, heart rate measurements showing in daily or weekly format.

Figure S14. The photographs showing the appearance changes of the mixture throughout the mixing process.

Figure S15. The custom-built multi-filament spinning system including a digitalized air pump allowing precise control of pressure level connected to a pressure regulating valve, a coagulation bath filled with saturated NaCl solution and a winding machine.

Figure S16. Weaving loom for the WRCT fabric fabrication by interlacing warp yarns and weft yarns together, scale bar: 10cm.

Figure S17. Schematic design of the WRCT wearable wrist band.

Figure S18. Production Pattern for the WRCT wearable wrist band.

**Table S1:** Comparative overview of radiative cooling performance in recent textiles.

| **Fabrication method** | **Functional polymer** | **Substrate** | **Nanoparticles** | **Nanoparticle loaded** | **Mid-infrared emissivity** | **Solar reflectance** | **References** |
| --- | --- | --- | --- | --- | --- | --- | --- |
| Coating | Cellulose triacetate | Cotton fabric | SiO₂ | Yes | 0.96 | 0.76 | [1] |
| Coating | PE | PE nonwovens | SiO₂ | Yes | 0.94 | 0.91 | [2] |
| Coating | PVDF-HFP | Cotton wipes | SiO₂ | Yes | 0.95 | 0.90 | [3] |
| Coating | PVDF-HFP | Cotton yarn | SiO₂ | Yes | 0.98 | 0.91 | [4] |
| Coating | PVDF-HFP, PDMS | Cotton textile | Nil | No | 0.95 | 0.94 | [5] |
| Electrospinning | Cellulose acetate | Cotton yarn | Al_2_O_3_ | Yes | 0.99 | 0.85 | [6] |
| Electrospinning | PVDF-HFP | Nil | TiO_2_, Al_2_O_3_ | Yes | 0.98 | 0.95 | [7] |
| Electrospinning | PVDF-HFP, PA6 | Nil | Nil | No | 0.97 | 0.94 | [8] |
| Electrospinning | PVDF-HFP, PAN | Nil | SiO₂, TiO_2_, Al_2_O_3_ | Yes | 0.95 | 0.93 | [9] |
| Electrospinning | PEO | Nil | Nil | No | 0.78 | 0.96 | [10] |
| Electrospinning | TPU | Nil | BN-nanosheet | Yes | 0.93 | 0.95 | [11] |
| Multi-layer laminating | HDPE, PVDF | HDPE fabric | BN | Yes | 0.99 | 0.93 | [12] |
| Multi-layer laminating | PLA, PEG, Cellulose fibers | Nil | SiO₂ | Yes | 0.98 | 0.87 | [13] |
| Multi-layer laminating | PLA, PTFE | PLA/ TiO_2_ fabric | TiO_2_ | Yes | 0.95 | 0.92 | [14] |
| Multi-layer laminating | PMP | Wool fabric | Silver nanowire | Yes | 0.85 | 0.97 | [15] |
| Wet spinning | Cellulose | Nil | SiO₂ | Yes | 0.98 | 0.94 | [16] |
| **Wet-spinning** | **PVDF** | **Nil** | **Nil** | **Nil** | **0.96** | **0.96** | **This work** |

Abbreviations list: HDPE = High-density polyethylene; PA6 = Polyamide 6; PAN = Polyacrylonitrile; PEO = Polyethylene oxide; PEG = Polyethylene glycol; PLA = Polylactic acid; PMP = Polymethylpentene; PDMS = Polydimethylsiloxane; PTFE = Polytetrafluoroethylene; PVDF = Polyvinylidene fluoride; PVDF-HFP = Poly(vinylidene fluoride-co-hexafluoropropylene); TPU = Thermoplastic polyurethane

Table S2. The fabric specification of WRCT fabric.

| **Fabric Specification** | |
| --- | --- |
| Fabric material | 100% PVDF |
| Fabric structure | Woven fabric |
| Fabric thickness | ~0.4 mm |
| Fabric weight | 133 g/m² |
| Warp density | 8 yarns/cm |
| Weave type | 1 × 1 plain weave |
| Weft density | 7 yarns/cm |

Video S1. Simulated heat flux distribution of Joule heating with unilateral heat source application

**Reference**

[1]	M. Chen, X. Zhao, and Y. Liu, "Dry and cool: A Janus coated textile for simultaneous radiative cooling and unidirectional sweat transport," *Progress in Organic Coatings,* vol. 211, p. 109794, 2026/02/01/ 2026.

[2]	Y. Zhai *et al.*, "Multifunctional Polyethylene Fiber Materials with Passive Radiative Cooling and Photocatalytic Antibacterial Properties," *Advanced Functional Materials,* vol. n/a, no. n/a, p. e29127, 2025/12/31 2025.

[3]	X. Yang, W. Qu, W. Tong, and B. Zhang, "Multifunctional Cooling Textiles with Enhanced Radiative and Moisture Management by One-Step Phase Separation," *ACS Applied Materials & Interfaces,* vol. 17, no. 21, pp. 31442-31453, 2025/05/28 2025.

[4]	C.-Q. Ma *et al.*, "Scalable and Sustainable Superhydrophobic Cooling Metacotton," *Advanced Fiber Materials,* vol. 7, no. 1, pp. 144-155, 2025/02/01 2025.

[5]	S. Zhang, F. Zhang, J. Su, and J. Han, "Hierarchical porous P(VDF-HFP)/PDMS coating enabled flexible textiles with passive daytime radiative cooling, super-hydrophobicity and durability," *Colloids and Surfaces A: Physicochemical and Engineering Aspects,* vol. 726, p. 137867, 2025/12/05/ 2025.

[6]	M. Zheng *et al.*, "Core–Shell Yarn Woven Metafabric: Integrated Autonomous Sweat Transport and Radiative-Perspirative Cooling," *Advanced Fiber Materials,* vol. 8, no. 1, pp. 359-369, 2026/02/01 2026.

[7]	D. Thiyagarajan, S. Li, and B.-K. Lee, "Electrospun bilayered PVDF-HFP-TiO2/Al2O3 nanofiber membranes for high-performance passive radiative cooling," *Journal of Materials Chemistry A,* 10.1039/D5TA04422G vol. 13, no. 36, pp. 30235-30243, 2025.

[8]	Q. Feng *et al.*, "A scalable Janus electrospun metafabric for synergistic radiative and moisture-wicking cooling," *Colloids and Surfaces A: Physicochemical and Engineering Aspects,* vol. 727, p. 138193, 2025/12/20/ 2025.

[9]	D. Thiyagarajan, S. Li, and B.-K. Lee, "Fibrous Sphere Architectures for Efficient Daytime Radiative Cooling and Moisture Management," *Nano Letters,* vol. 25, no. 14, pp. 5609-5615, 2025/04/09 2025.

[10]	D. Li *et al.*, "Scalable and hierarchically designed polymer film as a selective thermal emitter for high-performance all-day radiative cooling," *Nature Nanotechnology,* vol. 16, no. 2, pp. 153-158, 2021/02/01 2021.

[11]	J. Dong *et al.*, "Stretchable Thermoplastic Polyurethane/Boron Nitride Nanosheet Fabrics with Highly Anisotropic Thermal Conductivity for Multi-scenario Passive Radiative Cooling," *Advanced Fiber Materials,* vol. 7, no. 3, pp. 841-852, 2025/06/01 2025.

[12]	X. Zhang *et al.*, "Bridging the gap: A Janus structure textile with synergistic radiative cooling and heat conduction for high-efficiency thermal management," *Composites Part B: Engineering,* vol. 314, p. 113441, 2026/04/01/ 2026.

[13]	K. Zhao *et al.*, "Calamus-leaf-inspired structural engineering, self-cleaning, and radiative cooling of trilayer textiles for advanced PPE," *Industrial Crops and Products,* vol. 241, p. 122853, 2026/03/01/ 2026.

[14]	S. Zeng *et al.*, "Hierarchical-morphology metafabric for scalable passive daytime radiative cooling," *Science,* vol. 373, no. 6555, pp. 692-696, 2021/08/06 2021.

[15]	R. Wu *et al.*, "Spectrally engineered textile for radiative cooling against urban heat islands," *Science,* vol. 384, no. 6701, pp. 1203-1212, 2024/06/14 2024.

[16]	Q. Li *et al.*, "Eco-Friendly Skin-Wrinkle-Inspired Micro-Nano Structured Cellulose Composite Fibers for Highly Efficient Daytime Radiative Cooling," *ACS Nano,* vol. 19, no. 39, pp. 34596-34605, 2025/10/07 2025.
